# Advances in the Pharmacological and Non-pharmacological Management of Non-motor Symptoms in Parkinson’s Disease: An Update Since 2017

**DOI:** 10.2174/1570159X20666220315163856

**Published:** 2023-06-15

**Authors:** Daniel J. van Wamelen, Katarina Rukavina, Aleksandra M. Podlewska, K. Ray Chaudhuri

**Affiliations:** 1 Department of Basic & Clinical Neuroscience, Institute of Psychiatry, Psychology & Neuroscience, Division of Neuroscience, King’s College London, London, United Kingdom;; 2 Parkinson Foundation Centre of Excellence at King’s College Hospital NHS Foundation Trust, London, United Kingdom;; 3 Department of Neurology, Radboud University Medical Center, Donders Institute for Brain, Cognition, and Behaviour, Centre of Expertise for Parkinson & Movement Disorders, Nijmegen, the Netherlands

**Keywords:** Parkinson's disease, non-motor symptoms, autonomic symptoms, neuropsychiatric symptoms, pain, treatment, non-pharmacological treatment

## Abstract

**Background:**

Non-motor symptoms (NMS) are an important and ubiquitous determinant of quality of life in Parkinson’s disease (PD). However, robust evidence for their treatment is still a major unmet need.

**Objective:**

This study aimed to provide an updated review on advances in pharmacological, non-pharmacological, and exercise-based interventions for NMS in PD, covering the period since the publication of the MDS Task Force Recommendations.

**Methods:**

We performed a literature search to identify pharmacological, non-pharmacological, and exercise-based interventions for NMS in PD. As there are recent reviews on the subject, we have only included studies from the 1^st^ of January 2017 to the 1^st^ of December 2021 and limited our search to randomised and non-randomised (including open-label) clinical trials.

**Results:**

We discuss new strategies to manage NMS based on data that have become available since 2017, for instance, on the treatment of orthostatic hypotension with droxidopa, several dopaminergic treatment options for insomnia, and a range of non-pharmacological and exercise-based interventions for cognitive and neuropsychiatric symptoms, pain, and insomnia and excessive sleepiness.

**Conclusion:**

Recent evidence suggests that targeted non-pharmacological treatments, as well as some other NMS management options, may have a significant beneficial effect on the quality of life and need to be considered in the pathways of treatment of PD.

## 1 INTRODUCTION

Non-motor symptoms (NMS) are universally present in people with Parkinson’s disease (PwP) with a major impact on quality of life (QoL) [1]. In his ‘Essay on Shaking Palsy’, James Parkinson recognised a range of NMS, including pain, dribbling of saliva, and sleep disturbances [2]. Nevertheless, NMS have been neglected for decades, and the importance of individual as well as the burden of NMS have only been realised in the last 20 years [3]. There is now strong evidence to suggest that the overall burden of NMS has an even greater impact on quality of life than motor symptoms, not just in advanced disease but also in premotor and early motor PD [4]. Therefore, adequate treatment of NMS in PwP could contribute to improving quality of life, as signposted in the 2019 MDS evidence-based review committee paper [5]. The barriers to performing high-quality trials to treat NMS of PD are wide, ranging from non-awareness of industry and licensing authorities regarding the importance of NMS in the lives of PwP to a “one size fits all” approach of clinicians in relation to treating PD and lack of a holistic, personalised medicine approach [3]. Additionally, the problem can also be related to the concept that Parkinson’s disease (PD) is a syndromic condition with great heterogeneity, while NMS presentations may overlap, particularly at later stages of the disease [6-8]. Therefore, recent approaches have also taken into account non-motor subtype-specific treatment strategies as part of personalised medicine delivery [6]. In the current review, we aim to provide an overview of the evidence that has emerged since 2017 on NMS treatment in PwP, following previous reviews [5], including a discussion on certain developments that are likely to make their way into clinical practice in the future.

## 2 METHODS

We performed literature searches in electronic databases, including Medline and Cochrane Library, and checked references of included manuscripts to identify further relevant studies. We have included all pharmacological, surgical, and non-pharmacological studies describing treatments for NMS in PwP, regardless of whether the NMS was a primary or secondary outcome in the study. As extensive previous reviews have been published on the topic of NMS treatment in PwP, in order to reduce overlap with previous reviews, we have only included studies from the 1^st^ of January 2017 up to the 1^st^ of December 2021. We have limited the review to (randomised) clinical trials and non-randomised (including open-label) studies where NMS have been measured by validated outcome measures, as outlined in section 3, and excluded case reports. To keep the search as broad as possible, we did not have a priori restrictions regarding the language in which a study was published.

As many studies described multiple non-motor outcomes with the same intervention, in order to describe these findings, we have in this review grouped NMS together based on the Non-Motor Symptoms Scale (NMSS) where possible [9]. For each group of symptoms that were the subject of therapeutic interventions, we have divided these interventions into 1) pharmacological, 2) non-pharmacological, and 3) exercise-based treatments. Moreover, to assess the quality and usefulness of each study, we used the Oxford Centre for Evidence-Based Medicine (OCEBM) levels of evidence [10]. Subsequently, Grade Practice Recommendations were used to give a treatment recommendation for each of the treatments described [10].

## 3 RESULTS

Using the above strategy, we identified over 100 studies focusing on the treatment of cardiovascular and autonomic symptoms, sleep, excessive daytime sleepiness and fatigue, neuropsychiatric symptoms, and pain (Table **1**). These studies will be described in the below sections.

## 4 GENERAL NON-MOTOR BURDEN

Examples of recent developments to improve overall NMS burden include a small cross-over trial of 16 PwP, treated with mucuna pruriens, which showed no improvement in the burden of non-motor symptoms, as measured by the NMSQ [11]. A phase II placebo-controlled, small (19 PwP) study on Nabilone, a synthetic analogue of tetrahydrocannabinol at a daily dose of two mg, reported an overall improvement of NMS burden after four weeks, as measured by MDS-UPDRS Part I [12]. However, the small sample size precluded meaningful interpretation for the use of this drug in clinical practice. Also, non-pharmacological treatment for overall NMS burden has not been widely reported, but a sham-controlled study of caloric vestibular stimulation in 33 PD patients reported that active treatment improved NMSS scores by over 40 points up to a period of over one month after the end of active treatment [13]. In addition, it was shown that at least 7.5 hours per week of combined physiotherapy, massage therapy, and speech and occupational therapy in an inpatient hospital setting could improve NMSS scores, including most of the individual domain scores of this scale [14]. Nonetheless, the exact combination of therapy and how many hours per week were dedicated to which therapy were not described in this study.

NMS dominate the prodromal stage of PD [3], and trials related to NMS in the early stages of PD are also emerging. For example, Mullin *et al.* examined the effect of Ambroxol, a cough linctus, influencing glucocerebrosidase activity in 8 PwP with GBA1 mutation and 9 PwP without a GBA1 mutation and reported improved motor scores. However, the non-motor burden worsened significantly over a 6-month period [15]. In another study, implanted peripheral nerve grafts into the substantia nigra in PwP undergoing deep brain stimulation surgery reported no improvement in non-motor symptoms, measured by the MDS-UPDRS part I [16]. Also, in an RCT of Hoehn and Yahr stage 2-3 PwP, no non-motor improvement (UPDRS part I) was seen after intermittent intra-putaminal glial cell line-derived neurotrophic factor [17]. Thus far, therefore, only a handful of new studies have addressed the NMS burden in PD using novel techniques, but neither NMSS nor MDS UPDRS part I showed any improvement.

Further well-designed and adequately sized studies with NMS as a primary outcome are needed to determine the usefulness of interventions aimed to improve these symptoms. Overall, evidence is currently limited, and no level 1 for the treatment of general NMS has emerged over the last years.

## 5 CARDIOVASCULAR AND AUTONOMIC SYMPTOMS

An overview of currently investigated treatments for cardiovascular and autonomic symptoms in PwP is provided in Table **2**.

### 5.1 Orthostatic Hypotension

Cardiovascular and autonomic symptoms are common among PwP. Especially, orthostatic hypotension (OH) and constipation are common, with overall prevalence rates ranging from 10% to 70% and 11 to 83%, respectively [18-20]. OH can be asymptomatic or symptomatic (light-headedness, dizziness, and falls when standing), is often untreated, and almost inevitably leads to disability. The treatment of OH has seen recent developments, and two RCTs since 2017 have reported beneficial effects of fludrocortisone and droxidopa, respectively. Droxidopa was associated with improvements in dizziness/light-headedness, visual disturbances, weakness, and fatigue scores, alongside an increase in upright systolic blood pressure compared to placebo [21]. These findings add further evidence to those previously reviewed, as Droxidopa has already been classified as efficacious and possibly useful and, therefore, level 1A evidence is now available for its use in treating OH in PD [5].

Lower level evidence is available for other treatments, *e.g.*, in a small RCT with 13 PwP where fludrocortisone showed a 37% improvement in diastolic blood pressure drop on the orthostatic challenge (level of evidence 2B), while pyridostigmine bromide had no effect on symptom severity related to orthostatic hypotension and quality of life with both interventions [22]. Finally, also Kanegusuku *et al.* provided level 2B evidence showing that in 30 PwP with Hoehn and Yahr stages 2 and 3, resistance exercises decreased normalised low-frequency components of heart rate variability and reduced systolic blood pressure fall during orthostatic stress [23].

### 5.2 Sialorrhoea

Sialorrhoea, and subsequent dribbling of saliva, is a common and disabling NMS in PwP and affects 19-29% of newly diagnosed PD patients [24, 25]. Data from a cohort of unselected PwP showed that saliva dribbling occurs in 37.2% of PwP irrespective of HY stage, gender, and disease duration [26].

Since 2017, several studies have confirmed the efficacy of salivary gland injections with botulinum toxin (BNT) for the treatment of sialorrhoea in PwP. Tiigimae and colleagues reported that in 12 PwP injected with BNT-A for treatment of sialorrhea, a significant decrease in salivary flow rate at 4 weeks was noted, and there was a significant increase in salivary levels of Lactobacilli, but not Streptococcus mutans [27]. Two subsequent large randomised controlled trials confirmed these findings, and both Rima botulinum 2500 U and 3500 U significantly reduced unstimulated salivary flow rate scores [28]. The preceding SIAXI study with 184 subjects showed that incobotulinumtoxin A reduced unstimulated salivary flow rate after one month compared to placebo, both at 75U and 100U. The prevalence of side effects was low, including dry mouth in up to 5.4% and dysphagia in up to 2.7% of participants [29]. Therefore, level 1A evidence is available for the use of Botulinum toxin injections to treat sialorrhoea in PD, and its use can be strongly recommended (Table **1**).

Another treatment with level 2B evidence, which has been investigated recently is oral dihydroergotoxine mesylate (2.5mg BID) reduced sialorrhoea scores, measured through the UPDRS sialorrhoea subscore and the Sialorrhea Clinical Scale for PD, in 20 PwP with a two-thirds response rate being defined as at least 30% reduction in sialorrhoea scores [30].

### 5.3 Other Autonomic Symptoms

Constipation is one of the earliest symptoms of PD and is commonly present in the prodromal stage with two different subtypes: slow transit and outlet constipation [31]. For the treatment of gastrointestinal symptoms, one new study on probiotics in PwP has been published, showing that multistrain probiotics in a once-daily capsule formulation for four weeks significantly increased the number of spontaneous bowel movements, as well as improvements in stool consistency and quality of life [32], providing level 2B evidence for its use in PD. For the same symptoms, at least in PwP with motor fluctuations, also the addition of Opicapone, a third-generation COMT-inhibitor, might have benefit as it was found to improve gastrointestinal and urinary domain scores of the NMSS after three months of treatment in a large open-label study [33]. This outcome, however, was not the primary objective of this study.

For the pharmacological treatment of other autonomic symptoms in PwP, such as sexual dysfunction and overactive bladder, there have been very few additions to those already described in the reviews by Seppi and colleagues [5] and Quarracino *et al.* [34]. There have been no new studies since 2017 for erectile dysfunction or urge incontinence, with limited evidence for the usefulness of solifenacin for the latter [5, 34]. For hyperhidrosis, as also recently evidenced by the review performed by Niemann and colleagues, no high-level evidence-based recommendation for its management in PwP can be made [35].

### 5.4 Discussion and Further Developments

Although we have identified a number of efficacious treatments for cardiovascular and autonomic symptoms in PwP, there is insufficient evidence for many interventions to make adequate conclusions for clinical use. Indeed, for several indications, further studies, preferably in the form of RCTs, are required. This is particularly true for gastrointestinal symptoms in PwP. On the other hand, level 1 evidence is available for the treatment of OH with Droxidopa and for sialorrhoea in the form of botulinum toxin injections to the salivary glands. Nonetheless, some treatments show promising results, even though currently insufficient evidence is available. This is, for example, the case with Opicapone for urinary and gastrointestinal symptoms and probiotics for constipation [32, 33]. Moreover, efforts are being made to identify further useful treatments for autonomic symptoms in PwP, especially in non-pharmacological treatments. Examples include Ampreloxetine, a norepinephrine reuptake inhibitor, which is currently under investigation for symptomatic orthostatic hypotension in PwP (NCT03829657), following a successful proof-of-concept Phase II study (NCT02705755), although no results are yet available [36]. Also, a large-scale randomised clinical trial of exercise focused on the effects of daily abdominal muscle exercises on autonomic function, including orthostatic hypotension (NCT03343574), is underway. Other trials are investigating the effects of leg muscle therapy on changes in blood pressure and orthostatic hypotension as a secondary outcome measure (NCT03900000), an elastic abdominal binder as a potential treatment for orthostatic hypotension (NCT04920552), and the effects of nicotine patches on OH (NCT02452125).

## 6 SLEEP/FATIGUE

Sleep dysfunction in PwP is an umbrella term comprising multiple symptoms and conditions, ranging from excessive daytime sleepiness (EDS) and insomnia to fatigue. The prevalence of EDS in PD patients is 2-3 fold higher than in the general population, with controlled studies showing EDS in 34-54% of PD patients compared to 16-19% in healthy subjects [37, 38]. In drug-naïve, early PD patients, the prevalence of EDS varies between 9.6%-50% at baseline, rising to 23.4%-70% after two and five years [25, 39, 40] and clearly suggesting a progressive pattern over time. Sleep onset and maintenance insomnia occurring in PwP show a wide range with 20-80% prevalence of poor sleep reported in PD cross-sectional subjective assessments, up to 59% prevalence based on physician interviews, and only 32% based on the International Classification of Sleep Disorders (ICSD)-II criteria [38]. The data related to its progression during the course of the disease are heterogeneous, and, unlike EDS, insomnia appears to have a slower rate of progression, and some studies found no change in the prevalence of insomnia over time [41-43]. RBD, which is considered a major prodromal feature of PD, has a prevalence estimate in PwP that ranges from 16 to 47% [44]. However, RBD does not always precede the motor stage of PD and may develop later during the course of the disease [44]. Finally, the prevalence of pathological/central fatigue in PwP has a broad range and is estimated between 33% and 70% [45]. Progression data on fatigue is not robust, and at diagnosis, 50.2% of patients may have demonstrable fatigue, rising to 57.2% after two years [39, 46], although fatigue severity (as measured by the Fatigue Severity Scale) remained stable [46].

An overview of currently investigated treatments for sleep and related problems in PwP is provided in Table **3**.

### 6.1 Pharmacological Treatments

Well-established pharmacological treatment for sleep disorders in PD with the most robust evidence is Rotigotine transdermal patch, with level 1A evidence and a strong recommendation for its use. Examples of recent studies include the PD0013 trial, in which, it significantly improved PDSS-2 scores with no worsening of ESS scores [47, 48]. A meta-analysis of 10 studies with 1,844 patients treated with Rotigotine supported these notions. Here, it was shown that, compared with placebo, Rotigotine significantly improved the sleep/fatigue domain of the NMSS while also improving the overall non-motor burden score [49].

Other treatments interacting with dopaminergic neurotransmission, both with level 2B evidence, include Rasagiline and Opicapone. In a study with 30 patients (2:1 randomisation to treatment vs. placebo), significant improvements in sleep maintenance after eight weeks of Rasagiline treatment were observed using polysomnography which showed a decrease in wake time after sleep onset and number of arousals in addition to improvements in daytime sleepiness as measured by the Epworth Sleepiness Scale [50]. The OPTIPARK study addressed the effectiveness and safety of Opicapone, a third-generation catecholamine-O-methyl-transferase inhibitor, in over 500 PwP with motor fluctuations and reported improvements of circa 15% in the sleep domain of the NMSS after three months of treatment [33].

Circadian interventions, such as melatonin given at four mg daily for eight weeks, on the other hand, were unable to improve REM sleep behaviour disorder in 30 PwP enrolled in a recent double-blind RCT [51]. This seems to be in line with another similarly sized cohort of PwP where melatonin 25 mg once daily failed to show an improvement in clock gene expression as measured in serum [52]. Given the negative results (evidence level 2A), the above is unlikely to lead to changes in the advice given by the Movement Disorders Society Evidence-Based Medicine Committee in 2019 for treating sleep disorders in PwP [5].

### 6.2 Non-pharmacological Treatments

Rutten and colleagues compared the effects of 10,000 lux bright light therapy and placebo (200 lux light) in 83 PwP in a double-blind RCT and reported no benefit on mood as measured by the Hamilton Depression Rating Scale, while subjective sleep quality (not measured by validated scales) improved significantly, along with total salivary cortisol secretion [53]. Videnovic and colleagues showed that, in 31 moderately advanced PwP, bright light therapy was able to significantly improve daytime sleepiness and sleep quality as captured by the Pittsburgh Sleep Quality Index and PDSS-2, along with self-reported improvements in sleep fragmentation and ease of falling asleep [54]. A third placebo-controlled RCT (16 PwP) was unable to confirm the improvement in daytime sleepiness (ESS scores), although a post hoc analysis of patients with severe sleepiness (ESS score >11) revealed a significant greater decrease in ESS scores after BLT than after placebo [55].

In another RCT comparing acupuncture versus sham acupuncture in 40 PwP, both the general Fatigue score of the Multidimensional Fatigue Inventory after 5 weeks of treatment along with the Geriatrics Depression Scale and Epworth Sleepiness Scale ESS remained stable with no improvement [56]. These findings are in line with previous recommendations by Seppi and colleagues that insufficient evidence is available for acupuncture as a treatment for fatigue in PwP [5].

### 6.3 Exercise-based Treatments

Since 2017, a few studies have been published on exercise-based treatments for fatigue in PwP. For example, eight weeks of yoga treatment in 15 PwP, compared to 12 PwP not receiving this intervention, improved Parkinson’s fatigue scale-16 scores [57]. Atan *et al.* reported that, in 35 moderate to advanced PwP, an exercise programme comprising 30 minutes of conventional rehabilitation, including stretching, strengthening, and balance exercises, followed by 30 minutes of treadmill exercise five days a week for six weeks improved fatigue by up to 65-80%. In addition, pain improved by nearly 90% when measured by the Nottingham Health Profile [58]. However, the level of evidence for these studies was 3B and 2B, respectively, and the use of these treatments can, therefore, not yet be advised for use in PD.

### 6.4 Discussion and Future Developments

In spite of some evidence for the treatment of sleep and related disorders in PwP, improving PD‐related sleep problems and daytime somnolence is usually complex due to the heterogeneity of their causes [44, 48, 59]. One of the first steps could be an attempt to optimise dopaminergic therapies in PwP to improve nocturnal symptom control as level 1 evidence is available for the use of the dopamine agonist Rotigotine (Table **3**). On the other hand, options that might seem more intuitive, such as the use of Melatonin, including its use for RBD, are currently not supported by high-level evidence (Table **3**). In relation to the latter, potential contributors should be identified and addressed, such as antidepressant medication, which has been associated with causing or worsening RBD [60]. For the treatment of EDS, more options have been explored, including an increased number of non-pharmacological studies (Table **3**). Nonetheless, for most explored options, either insufficient evidence is available or treatments seem inefficacious, whereas the use of BLT seems promising to treat EDS in PwP [54, 55].

Further developments are expected, although the field of sleep-related problems is currently receiving less attention than some other NMS. Some examples of currently ongoing studies include adenosine A2A receptors, most notably istradefylline, used for the treatment of motor fluctuations in PwP. Here, Jenner *et al.* showed that several small open-label studies had explored the potential utility of istradefylline in the management of non-motor symptoms, including sleep and mood disorders, although the evidence thus far is limited [61]. In addition, further trials on light therapy with blue light, thought to target the retinohypothalamic tract more specifically, are underway [62, 63].

## 7 NEUROPSYCHIATRIC SYMPTOMS

Depression can affect between 11% and 45% of patients with PD [64-68]. Within this population, 17% present with major depression, 22% with minor depression, and 13% with dysthymic disorder, with lower rates being reported in population-based studies [69, 70]. In PwP, depression appears persistent after diagnosis, and studies have reported that 50-55.6% who had major depression at baseline will still suffer from it after six months to one-year follow-up [71, 72]. Anxiety is commonly associated with depression, although one European study reported that anxiety might not be discussed in 39.6% of patients in a clinical setting [73]. In PD, about 25% of PD patients experience clinically relevant symptoms of anxiety [74] and may have a generalised anxiety disorder as specified by the Diagnostic and Statistical Manual of Mental Disorders [75]. Given the frequent co-occurrence of mood/anxiety, hallucinations, and cognitive measures as outcomes in interventional trials, these symptoms have been grouped together. An overview of interventions is provided in Table **4**.

### 7.1 Pharmacological Treatments

**Fig. (1):**
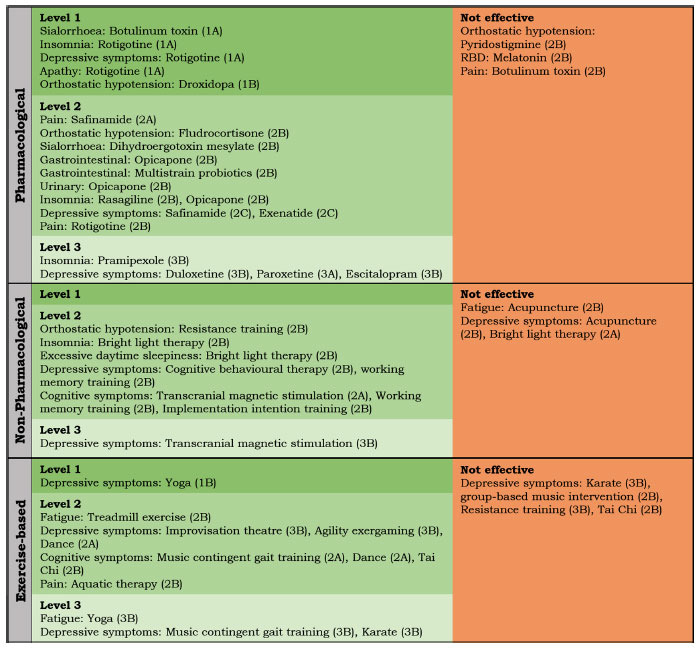
Recently investigated interventions for non-motor symptoms (published since 2017) in people with Parkinson’s disease. In green, effective treatments with a level of evidence, based on the Oxford Centre for Evidence-Based Medicine (OCEBM) levels of evidence [10], and in red, ineffective treatments with a level of evidence; references for interventions are provided in Table **1**. For a review of evidence-based non-motor interventions in Parkinson’s published up to and including 2016, see Seppi *et al.* 2018 [5].

Perhaps the most convincing evidence (level 1A) for the pharmacological treatment of depressive symptoms in PD is for Rotigotine. In a meta-analysis of 10 studies with 1,844 patients, compared to placebo, Rotigotine significantly improved apathy scores (Apathy Scale) and depressive symptoms (Beck Depression Inventory and the NMSS mood domain) [49]. Similarly, in relation to pharmacological treatment, in a posthoc analysis of two Safinamide trials, Cattaneo and colleagues showed that, compared to placebo, Safinamide was able to improve the PDQ-39 emotional well-being scores for two years since introduction, as well as improvement in Hamilton Depression Scale scores, in a cohort of over 400 PwP with motor fluctuations [76], however with absolute minor improvements. Ropinirole, on the other hand, had no effect on depression scores (measured by the Hamilton Depression Rating Scale) after seven weeks of treatment with 4mg in 32 PwP [77] (Table **4** and Fig. **1**).

In addition to this, studies have focused on antidepressant treatment in PwP, providing evidence ranging from level 3B to 2C. An example here includes a study involving 27 mildly to severely depressed PwP where both SSRIs (paroxetine and escitalopram) and duloxetine improved depression scores, measured by the Quick Inventory of Depressive Symptomatology scale [78]. Exenatide, a GLP-1 receptor agonist which is being trialed to examine potential disease-modifying effects, may also have a non-motor effect: Athauda and colleagues showed that, compared to placebo, PwP treated with exenatide-once weekly showed improvements in NMSS mood/depression symptoms after 48 weeks of treatment. In addition, an improvement was observed in the “emotional well-being” domain of the PDQ-39, but it was not sustained after three months. Interestingly, the mood/depression improvement occurred despite the lack of changes in motor scores, suggesting exenatide may exert independent effects on mood dysfunction [79]. Nonetheless, as these studies only provided lower level of evidence, none of the latter studies could be recommended for standard use in clinical practice (Table **4**).

One of the better studied pharmacological interventions for neuropsychiatric symptoms, with a level of evidence 2A, is pimavanserin, a selective serotonin 2A receptor inverse agonist. In PwP with psychosis, an analysis of a 6‐week randomised, double‐blind, placebo‐controlled, phase 3 trial of pimavanserin 34mg (equivalent to 40mg of pimavanserin tartrate) showed that in 185 PwP, it was well-tolerated and showed an improvement in PD‐adapted Scale for the Assessment of Positive Symptoms scores. Changes were most pronounced in those with additional cognitive impairment [80]. The open-label extension study additionally showed that the response was sustained at four and eight weeks [81, 82]. Moreover, preliminary evidence suggests that eight weeks of pimavanserin treatment might improve depression symptoms, reflecting improved Hamilton Depression Scale-17 scores in a single-arm, open-label study involving 45 PwP [83]. However, the adverse event rate of pimavanserin, at least in the clinical trials, was relatively high at almost 50% [82].

### 7.2 Non-pharmacological Treatments

Since 2017, three studies have focused on the effects of transcranial stimulation (TMS) in PwP, providing level 2B evidence for its use; the results, however, have been varying. One study showed that transcranial Direct Current Stimulation (tDCS) over the left dorsolateral prefrontal cortex in 22 PwP who received tDCS and computerised cognitive training for two weeks significantly reduced depressive symptoms and improved cognitive performances [84]. Another study with 16 PwP receiving remote DCS found preliminary evidence that this treatment could improve fatigue and cognitive processing speed, measured through the Wide Range Achievement Test 3 [85]. Others, however, found no effect on UPDRS part I depression scores after three months in 42 PwP [86]. As such, in addition to the conflicting results for TMS treatment in PwP, the conclusions were hampered by the small sample sizes.

Other treatments, with the level of evidence ranging between levels 3B and 2A, including cognitive behavioural therapy (CBT) and implementation intention training, have been investigated for use in PwP. Compared to verbal rehearsal, CBT improved self-reported depressive symptoms, but not anxiety, following a 10-week intervention period. In addition, the carer symptom burden was also reduced [87]. In a larger RCT with telephone-based CBT, weekly sessions for three months were able to improve depression, anxiety, and quality of life, measured by the Hamilton Depression Rating Scale, Hamilton Anxiety Rating Scale, and Medical Outcomes Study Short Form–36, respectively [88]. Similar treatments, such as implementation intention training, compared to verbal rehearsal, were able to stabilise memory scores (captured by the Prospective and Retrospective Memory Questionnaire Prospective Scale) after one month in 52 PwP. Interestingly, higher baseline depressive symptoms were found to be related to a better response to training in the intervention group [89]. Working memory training for 30 minutes three times a week for five weeks induced an improvement in working memory, measured by computerised outcomes, alongside an improvement in depression and apathy scores in 52 cognitively normal PwP [90]. Also, mindfulness-based interventions, such as stress reduction courses and those including cognitive therapy, significantly improved levels of depression, anxiety, and stress, measured by the Depression Anxiety and Stress Scale, short version (DASS-21), and Structured Clinical Interview for the DSM-IV, in 13 and 36 patients, respectively [91, 92] (Table **4** and Fig. **1**).

Several studies have also reported non-effective interventions for depression, anxiety, and cognitive performance. Since 2017, these include studies on bright light therapy with 10,000 lux light [55], acupuncture combined with bee venom [93], and group-based music intervention [94] (Table **4**).

### 7.3 Exercise-based Treatments

Over the last few years, multiple exercise-based interventions for neuropsychiatric symptoms have been explored in PwP, but not all were effective, such as a 12-week RCT of 60 minutes improvisation theatre sessions for anxiety and depression in 12 PwP [95]. Also, de Lima *et al.* were unable to demonstrate the effectiveness of resistance training in 33 PwP on depressive symptoms (Hamilton Depression Rating Scale scores) [96].

On the other hand, agility exergaming and stationary cycling showed beneficial effects (level of evidence 3B) in an RCT with improved Beck Depression Inventory scores [97]. Moreover, high cadence cycling showed an effect on emotional recognition, but there were no other changes in cognition or depression symptoms, among others measured through the Montreal cognitive assessment (MoCA) and Beck depression inventory (BDI) [98]. In a larger RCT with 138 PwP, a beneficial effect of yoga on Hospital Anxiety and Depression Scale scores, and especially anxiety scores, was reported [99]. Also, music-contingent gait (music play dependent on the user achieving a set stride length) training improved depression and anxiety (Geriatric Depression Score) in 30 PwP with no change in cognitive performance [100]. In addition, dance programmes were able to show an improvement in depression, apathy, and even MoCA scores, but not in fatigue scores [101, 102] (evidence level 2A), although some studies emphasise on Turo training, a form of dance-based exercise, showed no improvements in BDI scores, despite clear benefits on motor function [103]. Finally, Tai Chi was able to report improved MoCA scores in 41 PwP, along with an improvement in sleep scores, but not in depression and anxiety measures assessed by the Hamilton Depression Rating Scale [104], whereas karate had no effect in 19 PwP on mood, cognition, and overall wellbeing (measured by the Hospital Anxiety and Depression Scale) after twice-weekly session [105] (Table **1** and Fig. **1**).

### 7.4 Discussion and Future Perspectives

The neuropsychiatric symptoms in PwP are complex, and their underlying pathophysiology is heterogeneous, including dopaminergic, serotonergic, and noradrenergic systems [106]. Nonetheless, the traditional view has been to treat depressive symptoms in PwP in the same way as in people with major depressive disorder, and up to 25% of PwP are on an antidepressant at any given time, most commonly an SSRI [107, 108]. Interestingly, however, the evidence for the use of such antidepressant therapy is low, and levels of evidence are reflected in Table **4**. The best evidence for treating depressive symptoms in PwP is for Rotigotine, Pramipexole, and Venlafaxine (Table **4**). This would suggest that optimising dopaminergic treatment in PwP could be the first step when trying to address depressive symptoms. In fact, part of the depressive symptoms might stem from non-motor fluctuations, which often go unrecognised [109-111]. Evidence for the latter comes from the observation that aiming for continuous dopaminergic stimulation [112] improves especially mood and related symptoms [113, 114]. For non-pharmacological treatments, there is growing evidence, for *e.g.*, cognitive behavioural training, various forms of dance therapy, and transcranial magnetic stimulation (Table **4**). For apathy, less supporting evidence is available, and only in the form of pharmacological treatments, but especially Rotigotine appears efficacious [49] (Table **4**).

For treatment of cognitive dysfunction and related problems, much of the available evidence had already been reviewed elsewhere. For example, Seppi *et al.* showed that two large, randomised controlled cholinesterase inhibitor (ChEI) studies on PwP with dementia had been published, with one study showing a positive signal for rivastigmine and another with equivocal results for donepezil [5]. Moreover, the effects of ChEIs in PwP were classified as clinically modest, and the effects in mild cognitive impairment were even less pronounced. Of the recent newcomers in the treatment of cognitive dysfunction in PwP, especially the non-pharmacological and exercise-based treatments seem promising. For example, Transcranial magnetic stimulation [84, 85] and dance exercise [101, 102] seem to be useful interventions, although further level 1 evidence is needed before these could be used in standard clinical practice.

For psychosis, little has changed in terms of treatment advice over the last few years. The best way forward seems to consider dose reductions of dopaminergic drugs to a level that would lead to a resolution of psychotic symptoms while maintaining sufficient symptomatic motor control first [5]. Unfortunately, this might not always be a feasible approach, and further treatment might become necessary. Frequently, the treatment of psychosis in PD will include the addition of an antipsychotic agent, such as clozapine [5]. In recent years, however, more evidence is becoming available for pimavanserin, and considering its current level of evidence, it could be considered a treatment option for psychosis in PwP; however, it may not be readily available in all countries. In addition, there is some evidence to suggest that Pimavanserin causes an increased rate of hospitalisation in the first month after initiation and higher mortality within the first year of use [115]. Further dance-based studies focusing on the effects of ballet on NMS, including neuropsychiatric outcomes, are underway [116].

## 8 PAIN

### 8.1 Pharmacological Treatments

Among the recently explored non-oral pharmacological treatments for pain in PwP, Botulinum toxin A injections reduced numeric rating scale pain scores after 4 and 12 weeks of treatment, although not superior to placebo, perhaps related to the small number of patients (12) included in the study and the insufficiently enriched study population (evidence level 2B) [117]. On the other hand, several oral pharmacological treatments appear to be effective for analgesia in PwP. For example, Cattaneo *et al.* showed in a post-hoc analysis of pooled data from 352 PwP enrolled in two randomised trials that Safinamide, as add-on therapy in moderate to advanced PwP with stable doses of levodopa and motor fluctuations, after 24 weeks of treatment induced a reduction of the number of concomitant pain treatments of 23.6% compared to those receiving placebo (evidence level 1A). In addition, the PDQ-39 items related to musculoskeletal and neuropathic pain, as well as bodily discomfort, improved significantly [118]. These effects seemed to persist after two years of treatment, where the same authors showed that Safinamide improved the PDQ-39 items related to painful cramps/spasms and feeling unpleasantly hot or cold, as well as a significant reduction in concomitant pain treatments and a greater proportion of PwP not using analgesics [119]. Similar observations were made in a recent multi-center, prospective, open-label study enrolling 27 PwP with motor fluctuations; following a 6-month treatment with safinamide, significant improvement in KPPS total score and particularly scores for fluctuation-related pain was noted [120].

In a group of 93 PwP, of whom 70 completed the study, the introduction of Rotigotine improved the four 'affective dimension' items of the 12-item Pain Description List of the German Pain Questionnaire by 30% after at least 25 days of treatment. The study was, however, non-interventional, and the introduction of Rotigotine was based on standard clinical practice [121]. Also, Rascol and colleagues showed that the introduction of Rotigotine led to an approximately twofold numerical improvement in the King’s PD Pain scale domain “fluctuation-related pain” compared to placebo, but not on other aspects of pain [122]. Nonetheless, further confirmatory studies on the effect of Rotigotine on pain are needed, including studies focusing on pain as the primary outcome and deploying a randomised controlled study design [123].

### 8.2 Non-pharmacological and Exercise-based Treatments

Aquatic therapy (‘aquatic Ai Chi’) in twice-weekly sessions for 10 weeks, compared to exercise on dry land, was able to improve VAS scores for pain from 5.5 to 4.0 in 15 PwP, with no change in those doing exercise on dry land (5.8 pre-intervention and 5.3 post-intervention) [124]. This was the only exercise-based study (level of evidence 2B) that we have identified since 2017. No studies could be identified determining non-pharmacological treatments for pain published since the 1^st^ of January 2017, necessitating further dedicated research on this topic.

### 8.3 Discussion and Future Developments

Treatments for pain in PwP have been very recently reviewed by Rukavina *et al.*, who summarised that, to date, only three drugs (rotigotine, oxycodone/naloxone, and duloxetine) had been investigated specifically for their analgesic effects in PwP in double-blind RCT setting [125]. None of the studies met their primary endpoint [122, 126, 127]. In the current review, we were able to additionally identify safinamide as a possible treatment for pain, although these outcomes were not the primary outcome of the studies on which this evidence is based. As such, high-quality, robust evidence for pain management in PwP is lacking, but suggestions have been made to consider prolonged-release oxycodone/naloxone as a possibly useful treatment of chronic PD-related pain [5, 128]. Further evidence is needed, and some examples of studies that are currently underway to address pain in PwP include the OCEAN study, focusing on the effects of Opicapone on pain (https://www.clinicaltrialsregister.eu/ctr-search/trial/2020-001175-32/GB), and the CAN-PDP study evaluating the effects of cannabidiol (https://www.parkinsons.org.uk/research/can-pdp-cannabidiol-parkinsons-psychosis-0).

## 9 DEVICE-AIDED THERAPIES

The therapeutic effects of the device-aided therapies, including deep brain stimulation (DBS), apomorphine subcutaneous infusion (Apo), and intrajejunal levodopa infusion (IJLI), will be discussed separately due to their indications for use, limiting them to the more advanced stages of PD. In addition, the initiation of device-aided therapies is still determined by motor symptoms, especially uncontrollable motor fluctuations [129-131], although efforts are underway to include non-motor indications for these therapies, which are included, *e.g.*, in the Manage-PD tool [114, 132-134]. The use of device-aided therapies in PwP may also be limited by their relatively high costs [129]. An overview of the non-motor effects of device-aided therapies, all with few exceptions providing level 2B evidence for their use in PwP, is provided in Table **2**.

### 9.1 Deep Brain Stimulation

Several studies have shown an overall NMS burden improvement. Recently, Jost *et al.* showed that after 36 months of DBS-STN in 87 PwP, compared to 84 PwP on best medical treatment, significantly improved NMSS total scores with specific improvements in sleep/fatigue, urinary and miscellaneous NMSS domains [135]. Overall this study, as well as other studies, suggested that anterior, medial, and ventral STN-DBS were significantly associated with beneficial non-motor outcomes [136]. Similarly, after one month of DBS implantation, significantly greater improvement in NMSS total scores was seen in PwP after dorsolateral left-sided stimulation of the subthalamic nucleus, but not the ventromedial or dorsolateral positions on the right side [137]. In another study examining the effects of specific electrode positions in the STN in 50 PwP, it was shown that NMSS and quality of life improvements significantly depended on a more medial, anterior, and ventral neurostimulation, whereas activities of daily living and levodopa equivalent daily dose improvement depended on more posterior and lateral neurostimulation locations, respectively, with no differences in motor improvement between groups [136] (Fig. **1**).

Non-motor areas that have been specifically addressed in recent reviews and meta-analyses include mood, with an overall improvement in depressive and anxiety symptoms after STN-DBS [138, 139], and sleep disorders. In the latter, a meta-analysis of seven studies with 82 patients showed that STN DBS improved sleep quality and restless leg symptoms. However, the improvements depended on how sleep was scored in the individual studies [140]. After STN-DBS, the change to REM sleep behaviour disorder was less consistent, and no clear pattern of deterioration or improvement could be observed [141]. Other recent reviews on the non-motor effects of DBS in PwP include the one by Maheschwary on cognitive function, showing a declining trend in verbal fluency and attention domains of cognition, while other functions remained unchanged [142], and Bellini *et al.* who showed that mean bladder volumes at desire and urge point to void seemed to improve, as well as the quality of life associated with recurrent urinary tract infection, while urinary retention and incontinence seemed to increase [143]. Nonetheless, most included studies had small cohort sizes, and outcomes were not standardised. Also, pain appears to improve considerably after the initiation of DBS [144, 145]. Finally, since 2017, one study has focused on the effect of DBS on olfactory dysfunction in PwP. Here, it was shown that STN-DBS in 45 PwP significantly improved olfactory dysfunction one month and three months after implantation [146].

### 9.2 Subcutaneous Apomorphine Infusion

Prakash and Simuni [130] have recently reviewed the evidence for non-motor efficacy of subcutaneous apomorphine infusion, following a previous review by Rosa-Grilo *et al.* [147]. From the reviewed evidence, it can be generally concluded that this infusion therapy leads to improvements in swallowing, constipation, insomnia, restlessness, urinary dysfunction, and apathy. Moreover, especially the EuroInf study, confirmed by the EuroInf2 study that was published in 2019, has been very informative in regard to the non-motor effects of apomorphine, with both open-label studies showing an improvement in general non-motor burden, as well as sleep, fatigue, mood, gastrointestinal, and urinary symptoms [114, 132]. Aside from the EuroInf2 study, however, no new evidence on the non-motor effects of apomorphine has emerged since 2017. NMS outcome was also used during the TOLEDO study, which showed both short and medium-term motor efficacy of apomorphine, but NMS outcome data have not been published [148, 149].

### 9.3 Intrajejunal Levodopa Infusion

The non-motor effects of IJLI have been recently reviewed by Prakash and Simuni [130] and by Antonini and colleagues [150]. In general, IJLI shows wide-ranging improvements in general NMS as well as specific NMS, such as sleep, mood, pain, and gastrointestinal function [150]. Other studies include the GLORIA registry, the interim analysis of the Duoglobe study (with 54 centres in 10 countries), the EuroInf study, and, more recently, in 2019, the EuroInf2 study, showing improvements in cardiovascular symptoms, sleep and fatigue, mood, gastrointestinal, and urinary symptoms [114, 132, 151, 152], along with clear improvements in quality of life [152, 153]. Additionally, Kulisevsky and colleagues recently reported that IJLI improved anxiety scores in PwP with motor fluctuations and improved mood and verbal fluency, particularly during the first few hours of the infusion curve [154]. In another study, with 39 PwP in whom IJLI was initiated, NMSS total score improved, alongside six of the NMSS domains (sleep/fatigue, attention/memory, gastrointestinal tract, urinary, sexual function, and miscellaneous) after three months with maintained improvement after four years, except for the urinary domain [155]. Finally, this broad improvement seems to be confirmed by Honig *et al.*, demonstrating improvements in six of nine NMSS domains [156]. However, the INSIGHTS study showed comparable improvements in NMS with IJLI and optimal medical therapy in a randomised phase 3b open-label study, suggesting that these improvements are linked to Levodopa (https://clinicaltrials.gov/ct2/show/NCT02549092). As such, further improvements might be achieved by adding COMT-inhibitors to IJLI [157], and such results are awaited from Lecigon, a novel combination of IJLI with Entacapone [158].

### 9.4 Discussion

Device-aided therapies are now established worldwide for the management of advanced Parkinson’s disease, but the emphasis remains on the motor profile of PwP, even though NMS have also been shown to play a part in the prognostic aspects of the successful delivery of these therapies [134]. Considering the differential effect on NMS of these device-aided therapies, NMS might provide an additional guide to the delivery of personalised medicine in the advanced stage of the condition. However, given the current level of evidence, this stage has yet to be reached, and furthermore, the primary and currently only indication for the initiation of device-aided therapies remains uncontrollable motor fluctuations [129, 134]. Moreover, due to associated costs and feasibility [129], these therapies will not be within the primary field of therapeutic management in PwP.

## CONCLUSION

The present review summarises the available randomised and non-randomised clinical trials based on evidence published since 2017 regarding the treatment of NMS in PwP, including pharmacological, non-pharmacological, and exercise-based trials. Although we have been able to identify a number of efficacious treatments, most treatments and treatment strategies described here do not exceed level 2 evidence. Therefore, it is unlikely that such treatments will be widely used in clinical practice at this moment in time. Nonetheless, it is encouraging to see that NMS are being increasingly used as an endpoint in studies and not just as a secondary outcome measure but primary in some cases with the increasing use of NMSS, a robust, validated outcome tool since 2007 [9]. A further complicating matter is that a relatively large number of studies have based their results regarding NMS treatment on fairly small patient cohorts, necessitating further research, especially to address largely unexplored areas of treatment, such as sexual dysfunction, anxiety, fatigue, pain with its distinctive subtypes, and olfactory dysfunction in PwP, in addition to the problem posed by the increasing burden of NMS as the disease progresses [1]. Moreover, although interest in treatment for NMS in PwP is increasing, reflected by an increasing number of randomised and non-randomised studies including these symptoms as outcome measures or even as a primary outcome, many key non-motor areas still lack adequate and high-grade evidence for treatment. Here, we have reviewed the evidence of NMS treatment in PwP that has emerged since 2017. Nonetheless, this area is constantly changing, and (systematic) reviews generally have a half-life of only five years [159, 160]. Further and continued efforts are, therefore, needed, not only in providing evidence for NMS treatment but also in keeping (systematic) reviews up-to-date.

Compared to previous reviews and meta-analyses on the topic of NMS treatment in PwP, we have not been able to identify many novel pharmacological candidates. Many of the pharmacological treatments described in the current review had already been described up to 2017 [5], but the evidence for some of these treatments has been strengthened since then. Examples include treatments for neuropsychiatric symptoms (in particular for depressive symptoms) [78], including a Rotigotine transdermal patch with additional tentative indications as a useful treatment of pain and circadian abnormalities [47, 121, 161], and several randomised studies confirming the usefulness of botulinum toxin injections into the salivary glands for the treatment of sialorrhoea [27-29] (Fig. **1**). New molecules in pharmacological treatments for NMS in PwP, although further evidence for their usefulness is needed, include Opicapone, Safinamide, Exenatide, and possibly Istradefyline [33, 61, 76, 79, 118, 119].

## Figures and Tables

**Table 1:** Non-motor symptoms in people with Parkinson’s disease, for which (recent) treatments, are covered in this review.

Autonomic symptoms Constipation Orthostatic hypotension Sialorrhoea	Sleep and related disorders Insomnia REM sleep behaviour disorder Excessive daytime sleepiness Fatigue
Neuropsychiatric symptoms Depressive symptoms Apathy Cognitive symptoms Psychosis	Pain

**Table 2:** Interventions for cardiovascular and autonomic non-motor symptoms in people with Parkinson’s disease.

**Non-Motor Symptom**	**Intervention**	**Efficacy**	**Level of Evidence**	**Practice Implications**
Orthostatic hypotension	Droxidopa [5, 21]	Efficacious	1B	Recommended
	Fludrocortisone [22]	Possibly efficacious	2B	Option
	Ampreloxetine [36]	Possibly efficacious	2B	Option
	Pyridostigmine [22]	Likely inefficacious	2B	Do not use
	Resistance exercise [23]	Possibly efficacious	2B	Option
Sialorrhoea	Botulinum toxin injections [27-29]	Efficacious	1A	Recommended
	Dihydroergotoxine mesylate [5]	Possibly efficacious	2B	Option
	Ipratropium Bromide Spray [5]	Insufficient evidence	3B	Investigational
	Glycopyrrolate [5]	Likely efficacious	2B	Option
Gastrointestinal	Probiotics [32]	Possibly efficacious	2B	Option
	Opicapone [33]	Insufficient evidence	2B*	Do not use
Urinary symptoms	Opicapone [33]	Insufficient evidence	2B*	Do not use

* Not the primary outcome of the study

**Table 3:** Interventions for sleep and related symptoms in people with Parkinson’s disease.

**Non-Motor Symptom**	**Intervention**	**Efficacy**	**Level of Evidence**	**Practice Implications**
Insomnia	Rotigotine [49]	Efficacious	1A	Recommendation
	Continuous positive airway pressure^+^ [5]	Likely efficacious	2B	Option
	Rasagiline [50]	Likely efficacious	2B	Option
	Opicapone [33]	Insufficient evidence	2B*	Do not use
	Bright light therapy [53, 54]	Insufficient evidence	2B*	Do not use
	Controlled‐release formulation of levodopa/carbidopa [5]	Insufficient evidence	3B	Do not use
	Pergolide [5]	Insufficient evidence	3B	Do not use
	Piribedil [5]	Insufficient evidence	3B	Do not use
	Eszoplocine [5]	Insufficient evidence	3B	Do not use
	Melatonin [5]	Insufficient evidence	2B	Do not use
	Pramipexole [63]	Insufficient evidence	3B	Do not use
REM sleep behaviour disorder	Melatonin [51,52]	Likely inefficacious	2A	Do not use
Excessive daytime sleepiness	Bright light therapy [54,55]	Likely efficacious	2B	Option
	Modafinil [5]	Insufficient evidence	3B	Do not use
	Caffeine [5]	Insufficient evidence	3B	Do not use
	Continuous positive airway pressure^+^ [5]	Likely efficacious	2B	Option
Fatigue	Treadmill exercise [58]	Likely efficacious	2B	Option
	Acupuncture [56]	Likely inefficacious	2B	Do not use
	Yoga [57]	Insufficient evidence	3B	Do not use

*Not the primary outcome of the study; ^+^ for people with Parkinson’s and chronic obstructive apnoea syndrome.

**Table 4:** Interventions for neuropsychiatric symptoms in people with Parkinson’s disease.

**-**		**Intervention**	**Efficacy**	**Level of Evidence**	**Practice Implications**
Depressive symptoms	Pharmacological interventions	Rotigotine [49]	Efficacious	1A	Recommendation
		Pramipexole [5]	Likely efficacious	2A	Option
		Venlafaxine [5]	Likely efficacious	2A	Option
		Exenatide [79]	Likely efficacious	2C (post-hoc)	Option
		Safinamide [76]	Likely efficacious	2C (post-hoc)	Option
		Nortriptyline [5]	Likely efficacious	2A	Option
		Desipramine [5]	Likely efficacious	2A	Option
		Citalopram [5]	Insufficient evidence	3B	Do not use
		Sertraline [5]	Insufficient evidence	3B	Do not use
		Paroxetine [5]	Insufficient evidence	3B	Do not use
		Fluoxetine [5]	Insufficient evidence	3B	Do not use
		Ropinirole [77]	Likely inefficacious	2B	Do not use
		Paroxetine [78]	Insufficient evidence	3A	Do not use
		Escitalopram [78]	Insufficient evidence	3B	Do not use
		Duloxetine [78]	Insufficient evidence	3B	Do not use
		Amitryptiline [5]	Insufficient evidence	3B	Do not use
		Pergolide [5]	Insufficient evidence	3B	Do not use
		Rasagiline [5]	Insufficient evidence	3B	Do not use
		Selegiline [5]	Insufficient evidence	3B	Do not use
		Moclobemide [5]	Insufficient evidence	3B	Do not use
		Atomoxetine [5]	Insufficient evidence	3B	Do not use
		Nefazodone [5]	Insufficient evidence	3B	Do not use
	Non-pharmacological interventions	Cognitive behavioural training [87, 88]	Likely efficacious	2A	Option
		Working memory training [90]	Likely efficacious	2B	Option
		Transcranial magnetic stimulation [84]	Likely efficacious	2B	Option
		Bright light therapy [53, 55]	Likely inefficacious	2A	Do not use
		Acupuncture [93]	Inefficacious	2B	Do not use
		Mindfulness [91, 92]	Insufficient evidence	3A	Do not use
		Improvisation theatre [95]	Insufficient evidence	3B	Do not use
	Excercise-based interventions	Yoga [99]	Efficacious	1B	Recommendation
		Dance exercise [101-103]	Likely efficacious	2A	Option
		Group-based music intervention [94]	Likely inefficacious	2B	Do not use
		Tai Chi [104]	Likely inefficacious	2B	Do not use
		Agility exergaming [97]	Insufficient evidence	3B	Do not use
		Music contingent gait training [100]	Insufficient evidence	3B	Do not use
		Karate [105]	Insufficient evidence	3B	Do not use
		Karate [105]	Likely inefficacious	3B	Do not use
		Resistance training [98]	Likely inefficacious	3B	Do not use
Apathy	Pharmacological interventions	Rotigotine [49]	Efficacious	1A	Recommendation
		Piribedil [5]	Likely efficacious	2A	Option
		Rivastigmine [5]	Likely efficacious	2A	Option
Cognitive symptoms	Pharmacological interventions	Rivastigmine* [5]	Efficacious	1B	Recommendation
		Rasagiline [5]	Insufficient evidence	3B	Do not use
		Donepezil [5]	Insufficient evidence	3B	Do not use
		Galantamine [5]	Insufficient evidence	3B	Do not use
		Memantine [5]	Insufficient evidence	3B	Do not use
	Non-pharmacological interventions	Transcranial magnetic stimulation [84, 85]	Likely efficacious	2A	Option
		Working memory training [90]	Likely efficacious	2B	Option
		Implementation intention training [89]	Likely efficacious	2B	Option
	Exercise-based interventions	Dance exercise [101, 102]	Likely efficacious	2A	Option
		Music contingent gait training [100]	Likely efficacious	2B	Option
		Tai Chi [104]	Likely efficacious	2B	Option
		Karate [105]	Insufficient evidence	3B	Do not use
Psychosis	Pharmacological interventions	Pimavanserin [80-83]	Likely efficacious	2A	Option

* Only for dementia in people with Parkinson’s disease; for non-dementia cognitive impairment, there is currently insufficient evidence to recommend the use of Rivastigmine [5].

**Table 5:** Interventions for pain in people with Parkinson’s disease.

**Non-motor Symptom**	**Intervention**	**Efficacy**	**Level of Evidence**	**Practice Implications**
Pain	Safinamide [118,119]	Probably efficacious	2A*	Option
	Rotigotine [121]	Possibly efficacious	2B	Option
	Aquatic therapy [124]	Possibly efficacious	2B	Option
	Botulinum toxin injections [117]	Probably inefficacious	2B	Do not use

* Not the primary outcome of the study

**Table 6:** Summary of the non-motor effects of device-aided therapies (deep brain stimulation, continuous apomorphine infusion, and intrajejunal levodopa infusion), in manuscripts published since 2017, in people with Parkinson’s disease.

**-**	**DBS**	**Level of ** **Evidence^1^**	**Apomorphine ** **Infusion**	**Level of Evidence^1^**	**IJLI**	**Level of ** **Evidence^1^**
Overall non-motor burden	Overall burden [132, 135, 137]	2A	Overall burden [114, 130, 132, 147]	2A	Overall burden [114, 132, 151, 154]	2A
Neuropsychiatric symptoms	Depressive symptoms [138, 139]	2A	Depressive symptoms [114, 130, 132, 147]	2A	Depressive symptoms [114, 132, 151, 154]	2A
	Anxiety [138]	2A	Apathy [130, 147]	2A	Anxiety [114, 132, 151, 154]	2A
	Impulse control disorder [159]	2B*	-	-	-	-
Sleep	Insomnia [140]	2A	Insomnia [114, 130, 132, 147]	2A	Insomnia [114, 132, 151]	2A
	-	-	Fatigue [114, 132]	2A	Fatigue [114, 132, 151]	2A
Autonomic symptoms	Urinary symptoms [143]	2A	Urinary symptoms [114, 130, 132, 147]	2A	Urinary symptoms [114, 132, 151]	2A
	Constipation [143]	2A	Constipation [114, 130, 132, 147]	2A	Gastrointestinal symptoms [114, 132, 151]	2A
	Orthostatic hypotension [143]	2A	-	-	-	-
	Sialorrhoea [143]	2A	-	-	-	-
	Olfactory deficit [146]	2B	-	-	-	-

**Abbreviations:** DBS: deep brain stimulation; IJLI: intrajejunal levodopa infusion; 1: Based on the Oxford Centre for Evidence-Based Medicine (OCEBM), levels of evidence [10]; *: non-motor symptom reported was not the primary outcome in the study.
